# Environmental dependence of visible anthocyanin accumulation mediated by the *CmMYB6–CmbHLH2* complex

**DOI:** 10.3389/fpls.2026.1800973

**Published:** 2026-05-01

**Authors:** Qiwei Deng, Xiuting Zhu, Shuanglin Liu, Jishu Wang, Zhuo Zhou, Xiaoying Yu, Zhenkun Liao, Lu Xu, Yanlin Li, Lili Xiang

**Affiliations:** College of Horticulture, Hunan Agricultural University, Changsha, China

**Keywords:** anthocyanin, chrysanthemum, *CmMYB6-CmbHLH2*, darkness, drought, high-temperature

## Abstract

The anthocyanin biosynthesis pathway has been investigated as a candidate for visual reporting in plants, due to its pigment-producing capacity. Activation of this pathway is predominantly regulated by *MYB-bHLH* regulatory complexes. In a previous study, two chrysanthemum transcription factors, *CmMYB6* and *CmbHLH2*, were shown to induce anthocyanin accumulation when co-expressed. However, the stability and reliability of the visible pigmentation mediated by this complex under varying environmental conditions remain unclear. In this study, the visible anthocyanin phenotype was assessed under different environmental conditions, including darkness, high temperature, and drought stress. Our results show that anthocyanin accumulation mediated by the *35S::CmMYB6-CmbHLH2* complex is highly dependent on light and temperature. High temperature reduced anthocyanin accumulation mainly by decreasing transgene expression efficiency, whereas darkness suppressed pigmentation primarily at the post-transcriptional level, while drought stress moderately enhanced anthocyanin accumulation. Collectively, these findings indicate that environmental factors regulate anthocyanin accumulation mediated by the *CmMYB6-CmbHLH2* complex through distinct regulatory mechanisms, thereby suggesting that stable visible pigmentation mediated by the *MYB-bHLH* complex requires sufficient light intensity and a moderate temperature condition.

## 1 Introduction

Efficient identification of positive transformants remains a critical step in plant genetic transformation and functional genomics research (Yang et al., 2019; Arthikala et al., 2011). Conventional selection strategies mainly rely on antibiotic or herbicide resistance markers (Wang et al., 2024), which are time-consuming and may cause tissue damage or false positives. Visual phenotypes that allow direct, non-destructive observation of transgene expression therefore represent an attractive alternative for improving screening efficiency during plant transformation.

Among potential visual phenotypes, anthocyanin accumulation has attracted increasing attention due to its vivid pigmentation and endogenous origin in plants. Anthocyanins are water-soluble flavonoid pigments responsible for red to purple coloration in many plant tissues (Khoo et al., 2017). Because anthocyanin accumulation is generally non-toxic and can be detected by the naked eye without exogenous substrates or specialized equipment, it has been explored as a candidate visual indicator for monitoring gene expression and transformation events in plants (Khoo et al., 2017; Sendri and Bhandari, 2024).

Anthocyanin biosynthesis is predominantly regulated at the transcriptional level by *MYB-bHLH* regulatory complexes (Zhao et al., 2019), which coordinately activate the expression of key structural genes such as *CHS*, *DFR* and *ANS* (Gonzalez et al., 2008; Liu et al., 2018; Tai et al., 2013). Overexpression of *MYB* and *bHLH* transcription factors has been shown to effectively induce anthocyanin accumulation in a range of plant species, providing a visible phenotype that reflects transcriptional activation of the pathway (Li et al., 2022). Several studies have demonstrated that *MYB-bHLH* mediated anthocyanin induction can be used to mark transgenic tissues in crops and model plants, highlighting its potential utility as a visual marker (Liu et al., 2015; Xiang et al., 2021). However, whether anthocyanin accumulation driven by defined MYB-bHLH combinations can provide a robust and environment-independent visible phenotype remains unclear. Notably, most previous studies have focused on MYB-bHLH complexes from model plants (e.g., Arabidopsis, rice) or major crops, while the environmental stability of MYB-bHLH-mediated anthocyanin phenotypes from ornamental plants (such as chrysanthemum) has not been systematically explored, representing a key gap in the application of anthocyanin-based visual markers in ornamental plant genetic transformation.

In chrysanthemum, the transcription factors *CmMYB6* and *CmbHLH2* have been identified as positive regulators of anthocyanin biosynthesis (Xiang et al., 2021, Xiang et al., 2015; Tang et al., 2022). Co-expression of these two factors results in a strong effect on anthocyanin accumulation, leading to a clear red pigmentation phenotype in transformed tissues. This conspicuous coloration suggests that the *CmMYB6-CmbHLH2* complex has potential as a visual indicator for transgene expression. However, anthocyanin biosynthesis is highly sensitive to environmental factors, including light (Zhang et al., 2018; Liu et al., 2023; Maier and Ute, 2015; Shao et al., 2022; Chen et al., 2025; Wu et al., 2024), temperature (Kim et al., 2017; Chen et al., 2026; Zhang et al., 2018), and water availability (Ploenlap, 2015; Zhang et al., 2025; Xu et al., 2025; Zhang et al., 2023). Changes in these conditions can substantially alter anthocyanin accumulation through transcriptional, post-transcriptional, or metabolic regulation (Gao et al., 2021). As a result, the visible phenotype induced by *MYB-bHLH* complexes may vary across environments or plant species, limiting its reliability as a visual indicator. To date, how specific environmental factors influence anthocyanin accumulation driven by defined transcription factor combinations, such as *CmMYB6* and *CmbHLH2*, remains unexplored.

In this study, we evaluated the stability of visible anthocyanin accumulation mediated by the *35S::CmMYB6-CmbHLH2* complex under different environmental conditions, including darkness, high temperature, and drought stress. By combining phenotypic observation with anthocyanin content and gene expression analyses, we aimed to clarify how these environmental factors affect the anthocyanin-based coloration phenotype. This study not only provides the first systematic evaluation of the environmental adaptability of the CmMYB6-CmbHLH2-mediated visual phenotype, but also establishes a theoretical basis for optimizing anthocyanin-based visual marker systems in ornamental plants, offering new ideas and methods for the application of anthocyanin-based visual markers and providing a scientific basis for the rational use of such visual marker systems in plant genetic transformation.

## 2 Materials and methods

### 2.1 Plant material and treatment

The growing conditions of tobacco (*Nicotiana tabacum L*), ‘Manta’ rose and chrysanthemum fresh-cut flowers for the experiment were maintained in a growth chamber under the following conditions: temperature of approximately 25°C, relative humidity of about 60%, and a 16 h light/8 h darkness photoperiod. For transiently expressed tobacco treatment, Dark treatment were carried out a light intensity of 0 lx; Drought treatment were carried out by withholding water; High-temperature treatment was conducted at 37°C. Other conditions identical to the control.

### 2.2 Gene expression vectors and *Agrobacterium* strains

*Agrobacterium* strains GV3101 (AC1002S, Weidi, Shanghai, China) and EHA105 (AC1010S, Weidi, Shanghai, China) competent cells were purchased from Boyi Biotech. The recombinant plasmids were introduced into A. tumefaciens strains using the freeze-thaw method. Briefly, competent cells of GV3101 and EHA105 stored at -80°C were thawed on ice. Recombinant plasmids were added to the competent cell and gently mixed by tapping the tube. The mixture was incubated on ice for 5 minutes, followed by liquid nitrogen treatment for 5 minutes, a 37°C water bath for 5 minutes, and a final ice bath for 5 minutes. Subsequently, 700 μL of antibiotic-free LB medium was added, and the cells were incubated at 28 °C with a shaking at 200 rpm for 3 h. The cultures were centrifuged at 6000 rpm for 1 minute, and the supernatant was discarded and the pellet was resuspended. The suspension was spread onto LB agar plates containing appropriate antibiotics (50 mg/L kanamycin and 10 mg/L rifampicin) and incubated at 28 °C for 2 days. Single colonies were selected for PCR verification. Confirmed *Agrobacterium* cultures were mixed with an equal volume of sterile 50% (v/v) glycerol and stored at −80 °C for subsequent transient transformation experiments.

### 2.3 Transient transformation of *Agrobacterium* tobacco leaves

*Agrobacterium* cultures stored at -80°C were streaked onto LB solid medium containing 50 mg/L kan and 10 mg/L rif, and incubated at 28°C for 48 h. Single colonies were inoculated into 20 ml LB liquid medium with antibiotics and cultured at 28°C with a shaking at 200 rpm until the cultures reached the logarithmic growth phase. The bacterial cells were collected by centrifuge at 6000 rpm for 10 minutes, and resuspended in infiltration buffer containing 10 mmol/L MES, 10 mmol/L MgCl_2_, and 150 μmol acetosyringone (AS), adjusted to pH 5.6. The final bacterial suspension was adjusted to an OD_600_ of approximately 0.75. The healthy tobacco leaves were selected for infiltration. The *Agrobacterium* suspension was infiltrated into the tobacco leaves using a 1 mL disposable syringe without a needle. After infiltration, plants were incubated at 25 °C under a 16 h light/8 h dark photoperiod, with a light intensity of 8000 lx.

### 2.4 Measurement of total anthocyanin content

The content of total anthocyanin in flower petals was determined according to the method described by Akira Nakatsuka et al (Nakatsuka et al., 2008). The total anthocyanin content of tobacco leaves were measured based on the method described by Neff and Chory (Neff and Chory, 1998), with minor modifications. Briefly, 0.1 g of infiltrated leaves patch were weighted and ground into powder in liquid nitrogen. The powder was extracted with 300 μL of 1%(v/v) methanol solution and incubated in the dark at 4°C for 24 h. then 200 μL of distilled water was added to dilute the extract to a final concentration of 60% methanol, followed by the addition of an equal volume (500 μL) of chloroform. The mixture was vortexed thoroughly and centrifuge at 6000 rmp for 5 minutes. An aliquot of 400 μL of the supernatant was collected and diluted 1:1 with 60% methanol, and the absorbance was measured at 530 nm and 657 nm using a spectrophotometer. The relative total anthocyanin content (OD/gFW) was calculated using the formula:

Anthocyanin content (OD/g FW) = (A530 - A657)/M, M represents fresh weight of the sample (g).

### 2.5 RNA extraction and RT-qPCR

Total RNA was extracted from the infiltrated patch of tobacco leaves using a Plant RNA Extraction Kit (AG21019, Accurate Biology, Changsha, Hunan, China). First-strand cDNA was synthesized from the extracted RNA using the R323 Reverse Transcription Kit (R323-01, Vazyme, Naijing, Jiangsu, China) following the manufacturer’s protocol. RT-qPCR analysis was performed with SYBR Green dye (Q711-02, Vazyme, Nanjing, Jiangsu, China) using a real-time PCR system. Gene expression levels were calculated using the (Ct)2^−ΔCt^ method, with NtActin serving as the internal reference gene. Primer sequences are listed in **Table 1**.

**Table 1:** RT-qPCR primers.

Experiment	Primer name	Primer sequence (5’ - 3’).
Real-time qPCR	NtActin	F: AATGGAACTGGAATGGTCAAGGC R: TGCCAGATCTTCTCCATGTCATCCCA
	*CmMYB6*	F: ATGGGGGAGTACAGAAAAATG R: TCATAGTTGGTCCGAATTTA
	*CmbHLH2*	F: GGCTGCCAGCGGACCACCTCG R: GTAGTATCCATCTCCCCATACC

### 2.6 Data statistics and analysis

The experimental data were statistically analyzed by Excel2013, SPSS software and plotted by Graphpad Prism 9.5.

## 3 Results

### 3.1 *35S::CmMYB6-CmbHLH2* vector construction

**Figure 1:**
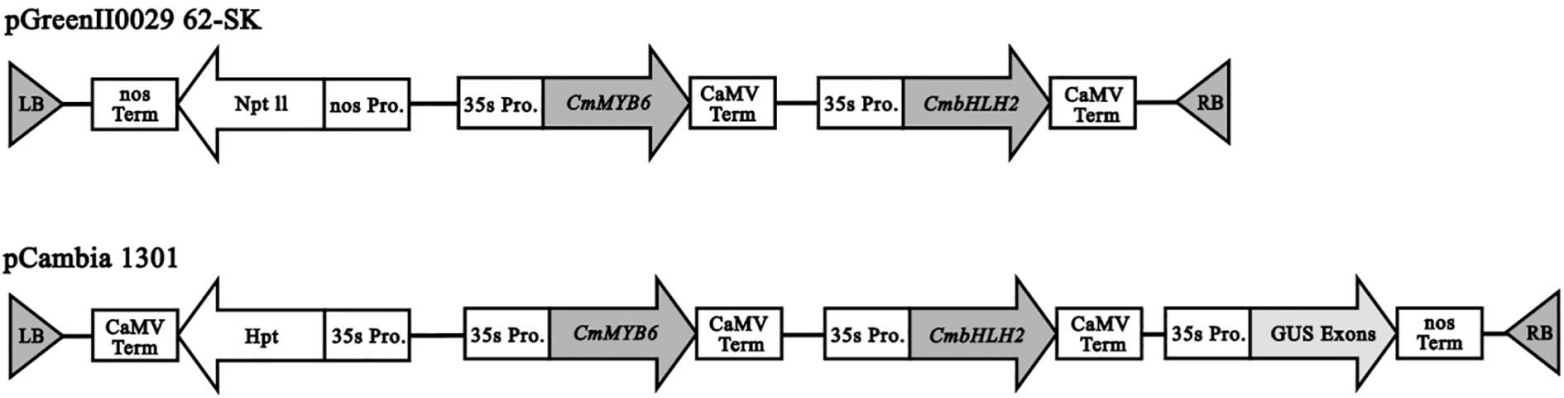
Diagram of *35S::CmMYB6-CmbHLH2* overexpression vector. LB, left boundary of T-DNA region; Nos Term, terminator of nopaline synthase gene; Npt II, Kanamycin resistance gene, used to screen successfully transformed plant cells; Hpt, hygromycin B phosphotransferase gene, providing resistance to hygromycin B; 35S Pro, Cauliflower Mosaic virus 35S promoter; CaMV Term, terminator of cauliflower Mosaic virus; *CmMYB6*, Gene of transcription factor regulating anthocyanin synthesis in Chrysanthemum; *CmbHLH2*, a transcription factor gene in Chrysanthemum that regulates anthocyanin synthesis; GUS Exons, Exons encoding β-glucuronidase (GUS).

Full-length coding sequences (CDSs) of *CmMYB6* and *CmbHLH2* genes were cloned from chrysanthemums cultivar ‘Jimba’, and then were integrated into the expression vectors pGreenII0029 62-SK and pCambia1301 respectively through multi-fragment homologous recombination, generating two *35S::CmMYB6-CmbHLH2* dual-gene expression vectors (**Figure 1**).

### 3.2 Effects of vector type and *Agrobacterium* strain on *CmMYB6-CmbHLH2* expression and anthocyanin accumulation

To evaluate the effects of different vector types and *Agrobacterium* tumefaciens strains on the overexpression efficiency of *CmMYB6-CmbHLH2*, the pGreenII0029 62-SK (SK) and pCambia1301 (1301) vectors were individually introduced into the GV3101 and EHA105 strains, and transiently expressed in tobacco leaves. The overexpression performance was assessed based on visible pigmentation, total anthocyanin accumulation, and target gene expression levels.

**Figure 2:**
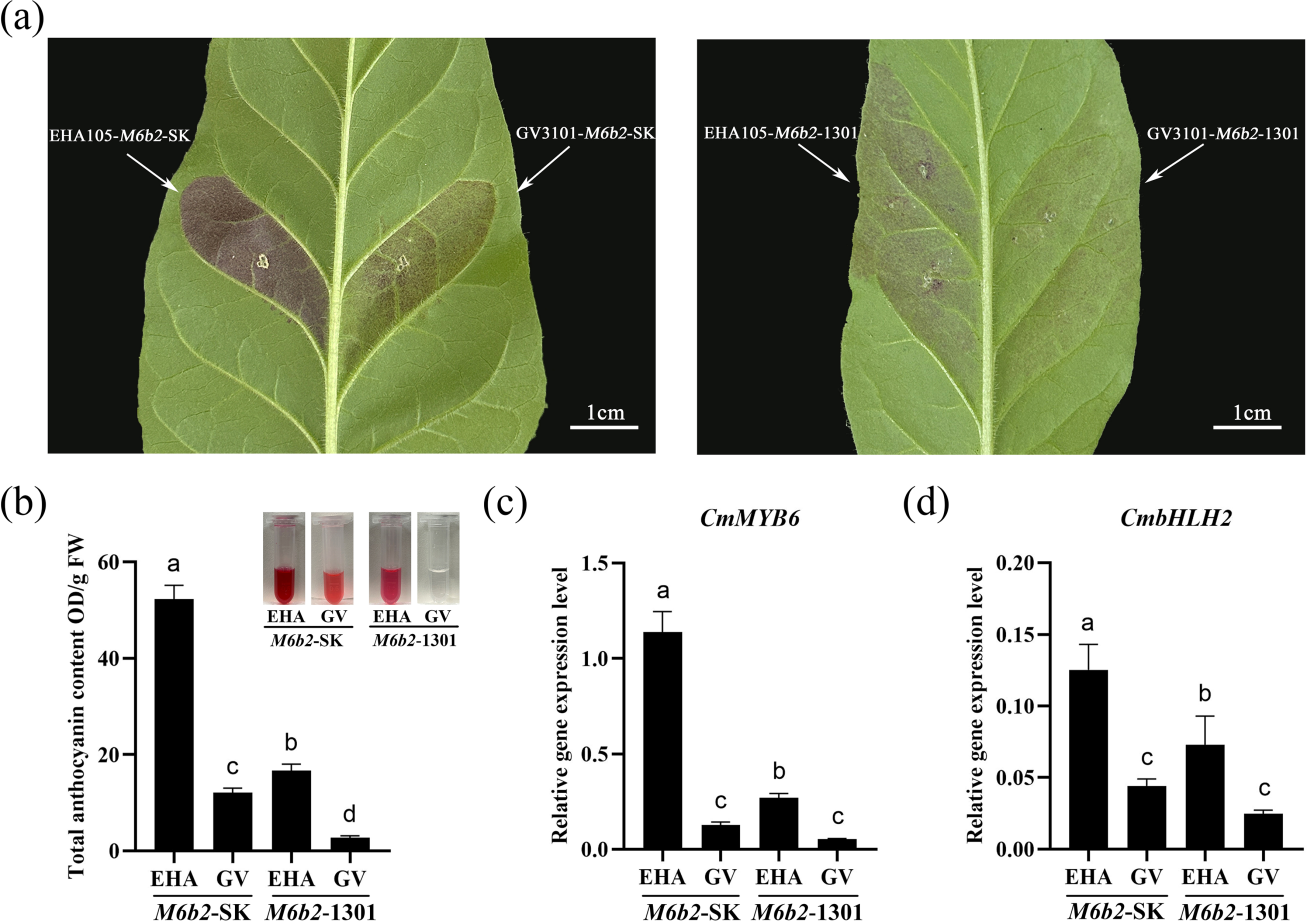
Transient expression of *CmMYB6-CmbHLH2* in tobacco leaves using different *Agrobacterium* strains and vectors. **(a)** Phenotypes of tobacco leaves overexpressed *CmMYB6-CmbHLH2* using different *Agrobacterium* strains and vectors; **(b)** Total anthocyanin content of tobacco leaves after overexpression; **(c)** Relative expression of *CmMYB6* in tobacco leaves; **(d)** Relative expression of *CmbHLH2* in tobacco leaves; *M6b2* is the abbreviation of *CmMYB6-CmbHLH2*; Different lowercase letters indicate significance differences at P< 0.05.

As shown in **Figure 2**, tobacco leaves infiltrated with the EHA105 strain carrying the SK vector (*35S::CmMYB6-CmbHLH2*-SK) exhibited the strongest red coloration phenotype (**Figure 2a**). Correspondingly, this combination resulted in the highest anthocyanin accumulation (52.23 OD/g FW) (**Figure 2b**). The EHA105 strain harboring the 1301 vector showed a moderate increase in anthocyanin content (16.66 OD/g FW), whereas both GV3101-based combinations displayed significantly lower anthocyanin levels, with the GV3101 strain carrying SK and 1301 vector had anthocyanin contents of 12.06 and 2.69 OD/g FW, respectively. However, none of the four empty vector (EV) controls exhibited a red coloration phenotype, and anthocyanin accumulation was barely detectable, with no significant differences among them (**Supplementary Figure 1**).

Consistent with anthocyanin accumulation, the expression levels of *CmMYB6* and *CmbHLH2* were highest in the EHA105-SK combination and gradually decreased in the other treatments (**Figures 2c, d**), indicating a strong positive correlation between gene expression and pigment accumulation.

Taken together, these results demonstrate that the EHA105 strain exhibits stronger infectivity and transgene expression capability than GV3101, and that the SK vector confers higher expression efficiency than the 1301 vector. Therefore, the SK vector combined with the EHA105 strain represents the optimal system for efficient overexpression of *CmMYB6-CmbHLH2* and subsequent anthocyanin induction.

### 3.3 Light is indispensable for visible anthocyanin accumulation mediated by *35S::CmMYB6-CmbHLH2*

To evaluate the coloration stability of the *35S::CmMYB6-CmbHLH2* complex under dark conditions, tobacco leaves transiently transformed into *35S::CmMYB6-CmbHLH2*-SK and the corresponding empty vector (EV) were treated with darkness and the normal light (8000 lx) as a control. Phenotypic coloration, total anthocyanin content, and the expression levels of *CmMYB6* and *CmbHLH2* were analyzed.

**Figure 3:**
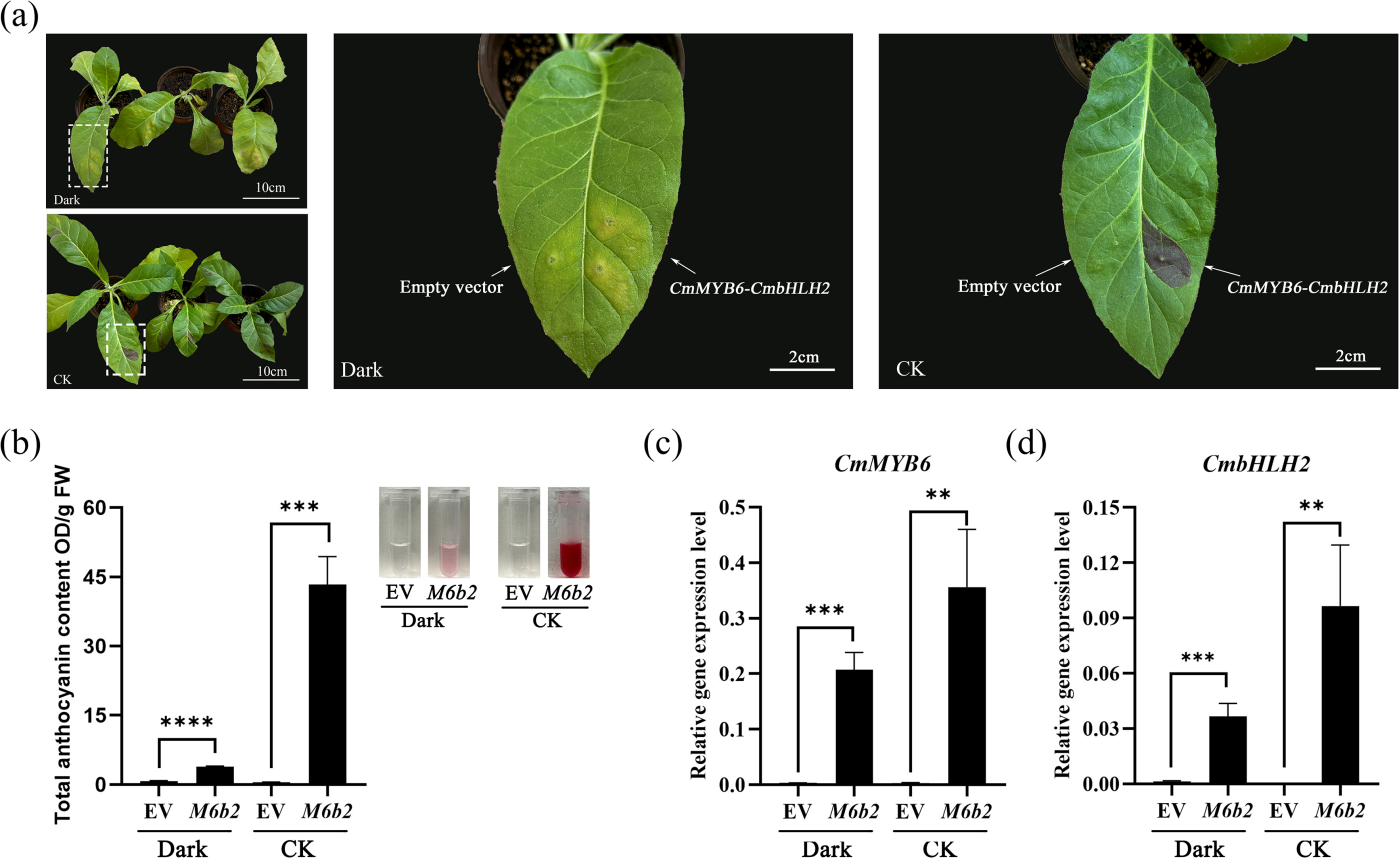
Comparison of the *35S::CmMYB6-CmbHLH2* transiently transformed tobacco leaves under dark and control conditions. **(a)** Phenotypic comparison of transiently transformed tobacco leaves under dark treatment and the control (CK); **(b)** Total anthocyanin content in transiently transformed tobacco leaves under dark treatment and the control (CK); **(c)** Relative expression of *CmMYB6* in transiently transformed tobacco leaves under dark treatment and the control; **(d)** Relative expression of *CmbHLH2* in transiently transformed tobacco leaves in the dark treatment and the control treatment group; Different lowercase letters indicate significance differences at P< 0.05; EV, Empty vector. ** indicates significant correlation at the P ≤ 0.01 level; *** indicates significant correlation at the P ≤ 0.001 level; **** indicates significant correlation at the P ≤ 0.0001 level.

Under dark treatment, leaves transformed with *35S::CmMYB6-CmbHLH2* showed only very faint red pigmentation with nearly no visible phenotype. In contrast, the same transformed leaves developed clear anthocyanin-associated red coloration under normal light conditions (**Figure 3a**). EV-infiltrated leaves exhibited no visible pigmentation under either treatment, ruling out background interference from the vector or Agrobacterium itself.

Consistent with the phenotypic observations, dark treatment caused a drastic reduction in total anthocyanin content in CmMYB6-CmbHLH2-transformed leaves: the content dropped from 43.31 OD/gFW in the control group to 3.82 OD/gFW, representing a 91% decrease (**Figure 3b**). Anthocyanin levels in EV controls remained at background levels under both treatments, indicating that the observed changes in anthocyanin content were not due to stress-induced endogenous responses, but specifically resulted from the CmMYB6-CmbHLH2 complex.

Further gene expression analysis showed that dark treatment significantly repressed the transcript levels of *CmMYB6* and *CmbHLH2*, leading to 42% and 62% downregulation, respectively (**Figures 3c, d**). Notably the magnitude of transcriptional repression was far lower than the reduction in anthocyanin content, suggesting that under dark conditions, anthocyanin biosynthesis mediated by the *CmMYB6-CmbHLH2* complex is likely regulated by post-transcriptional or protein-level regulatory mechanisms in addition to transcriptional control.

Overall, these results demonstrate that darkness severely impairs anthocyanin accumulation mediated by *CmMYB6* and *CmbHLH2*, resulting in the loss of visible pigmentation despite detectable expression of CmMYB6 and CmbHLH2. Thus, the *35S::CmMYB6-CmbHLH2* based visual reporter system is unstable under dark conditions and cannot reliably reflect transgene overexpression efficiency in the absence of light.

### 3.4 Effect of drought treatment on anthocyanin-based coloration mediated by *35S::CmMYB6-CmbHLH2*

To evaluate the coloration stability of the *35S::CmMYB6-CmbHLH2* complex under drought conditions, the tobacco leaves transiently transformed into *35S::CmMYB6-CmbHLH2-SK* and the corresponding empty vector (EV) were subjected to drought stress, with normally watered plants serving as controls. Phenotypic coloration, total anthocyanin content, and the expression levels of *CmMYB6* and *CmbHLH2* were analyzed to assess the reliability of the visible phenotype in reflecting transgene expression under drought conditions.

**Figure 4:**
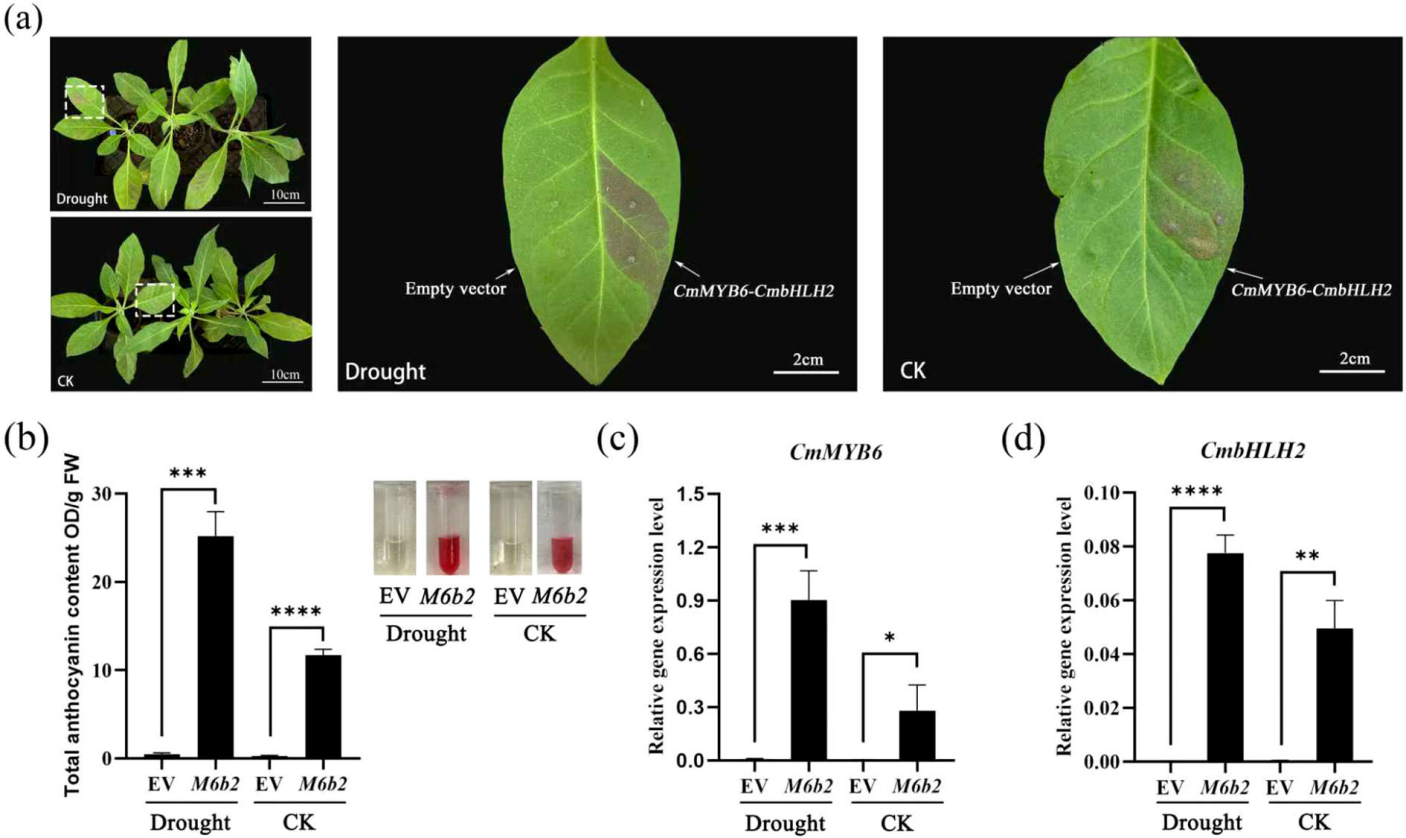
Comparison of the *35S::CmMYB6-CmbHLH2* transiently transformed tobacco leaves under drought and control conditions. **(a)** Phenotypic comparison of transiently transformed tobacco leaves between drought treatment group and control treatment group; **(b)** Total anthocyanin content of tobacco leaves transiently transformed *35S::CmMYB6-CmbHLH2* and the empty vector (EV) in drought treatment and control group; **(c)** Relative expression of *CmMYB6* in transiently transformed tobacco leaves under drought and control groups; **(d)** Relative expression levels of *CmbHLH2* in transiently transformed tobacco leaves under drought treatment and control; Different lowercase letters indicate significance differences at P< 0.05. EV, Empty vector. * indicates significant correlation at the P ≤ 0.05 level; ** indicates significant correlation at the P ≤ 0.01 level; *** indicates significant correlation at the P ≤ 0.001 level; **** indicates significant correlation at the P ≤ 0.0001 level.

As shown in **Figure 4a**, drought treatment induced a pronounced red pigmentation at the transformation sites, which was visibly stronger than that observed in the control group. In contrast, EV-infiltrated leaves exhibited no visible pigmentation under either drought or control conditions, ruling out background interference from the vector or Agrobacterium itself. Consistent with the phenotypic observations, total anthocyanin content in drought-treated leaves reached 25.19 OD/gFW, significantly higher than that in the control group (11.67 OD/gFW) (**Figure 4b**). Meanwhile, anthocyanin levels in EV controls remained at nearly undetectable background levels under both treatments, confirming that the observed changes in anthocyanin content were specifically attributed to the CmMYB6-CmbHLH2 complex rather than stress-induced endogenous responses.

Furthermore, transcript levels of *CmMYB6* and *CmbHLH2* were significantly upregulated under drought stress compared with the control (P < 0.05) (**Figures 4c, d**), indicating enhanced transcriptional activation of the anthocyanin regulatory module. By comparison, the expression of CmMYB6 and CmbHLH2 in EV-infiltrated leaves was barely detectable under both treatments.

The above results demonstrate that the *35S::CmMYB6-CmbHLH2* mediated pigmentation phenotype remains stable under drought conditions and that visible anthocyanin accumulation reliably reflects transgene expression efficiency in this condition.

### 3.5 High temperature suppresses anthocyanin accumulation mainly by reducing transgene expression

To evaluate the coloration stability of the *35S::CmMYB6-CmbHLH2* complex under high temperature conditions, the tobacco leaves transiently transformed into *35S::CmMYB6-CmbHLH2* and the corresponding empty vector (EV) were treated at 37 °C and used 25°C as the control.

**Figure 5:**
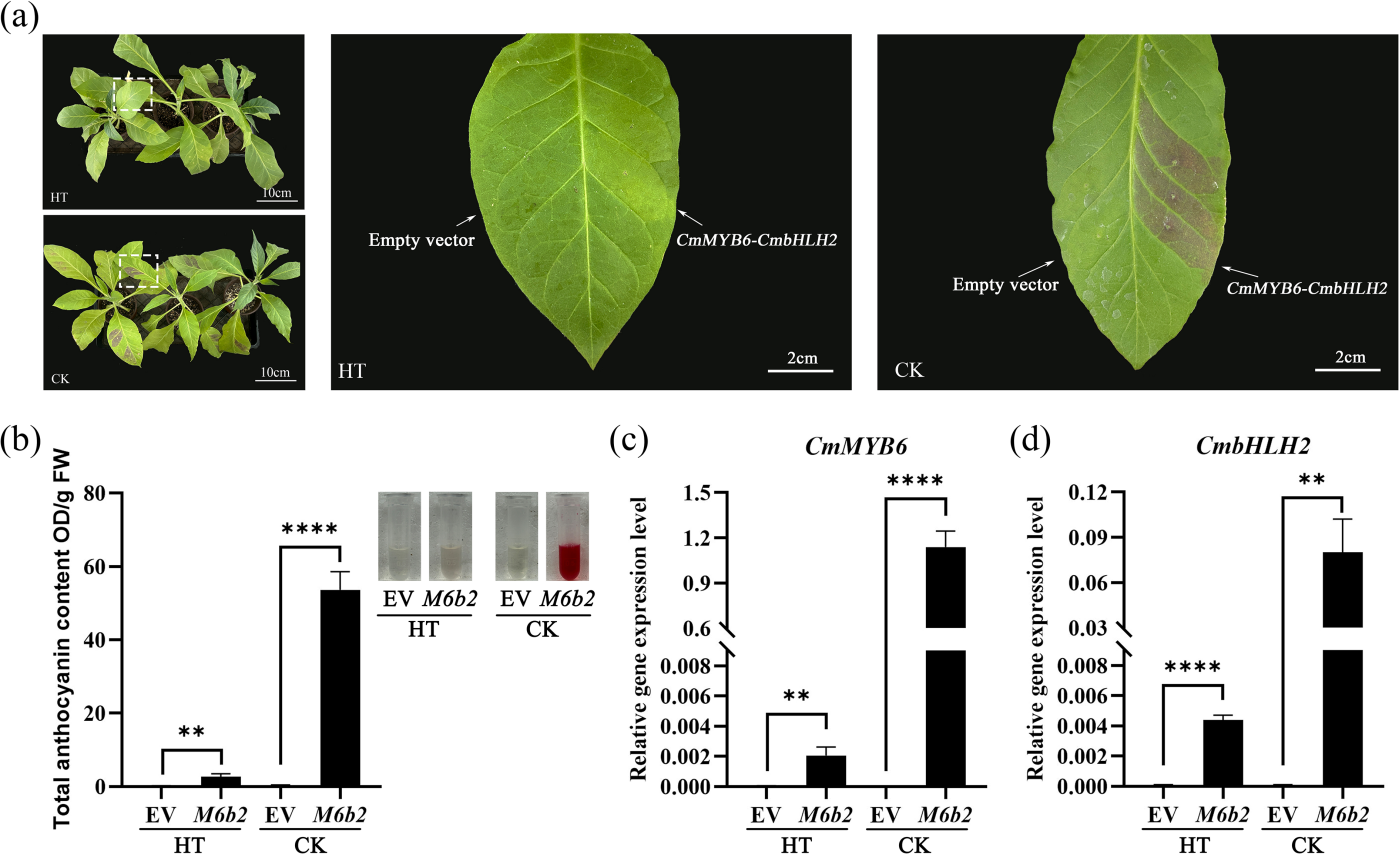
Comparison of the *35S::CmMYB6-CmbHLH2* transformed tobacco leaves under high temperature and control conditions. **(a)** Phenotypic comparison of tobacco leaves transiently expressing *35S::CmMYB6-CmbHLH2* under high-temperature treatment (37°C, HT) and the control condition (25°C, CK); **(b)** Total anthocyanin content in tobacco leaves expressing *35S::CmMYB6-CmbHLH2* and the empty vector (EV) under HT and CK conditions; **(c)** Relative expression of *CmMYB6* in overexpressed tobacco leaves under HT and CK conditions; **(d)** Relative expression of *CmbHLH2* in overexpressed tobacco leaves under HT and CK conditions; Different lowercase letters indicate significant differences at P < 0.05; EV, Empty vector; HT, High temperature. ** indicates significant correlation at the P ≤ 0.01 level; **** indicates significant correlation at the P ≤ 0.0001 level.

The results showed that no visible red pigmentation was observed at the infiltration sites under high temperature treatment (**Figures 3**-**5a**), whereas a pronounced red phenotype was observed in the control group. In contrast, EV-infiltrated leaves exhibited no visible pigmentation under either high temperature or control conditions. Consistently, anthocyanin quantification revealed that total anthocyanin content in the control group was 53.59 OD/gFW, while it was markedly reduced to 2.68 OD/gFW under high temperature, representing a decrease of approximately 95% (**Figure 5b**). Anthocyanin levels in EV controls remained at nearly undetectable background levels under both treatments, indicating that the observed changes in anthocyanin content were not due to high temperature-induced endogenous responses, but specifically resulted from the CmMYB6-CmbHLH2 complex.

Further analysis showed that the transcript levels of *CmMYB6* and *CmbHLH2* were strongly suppressed under high temperature conditions, with reduction of 99% and 95%, respectively, compared with the control (**Figures 5c, d**). Notably, their expression levels under high temperature were not significantly different from those of the empty vector control (CK) (P>0.05). By comparison, the expression of CmMYB6 and CmbHLH2 in EV-infiltrated leaves was barely detectable under both high temperature and control treatments, verifying that the detected transcriptional changes were derived from the transgene rather than endogenous tobacco homologs.

Collectively, these results indicate that high temperature severely impairs the visible anthocyanin phenotype mediated by the *CmMYB6-CmbHLH2* complex, primarily by inhibiting transgene expression, thereby resulting in a dramatic reduction in anthocyanin accumulation.

### 3.6 Evaluation of *CmMYB6–CmbHLH2*-mediated anthocyanin induction in different ornamental plant species

To assess the versatility of the *35S::CmMYB6-CmbHLH2* complex in inducing anthocyanin accumulation across ornamental plants, we performed transient expression assays in rose (*Rosa hybrida*) and chrysanthemum.

**Figure 6:**
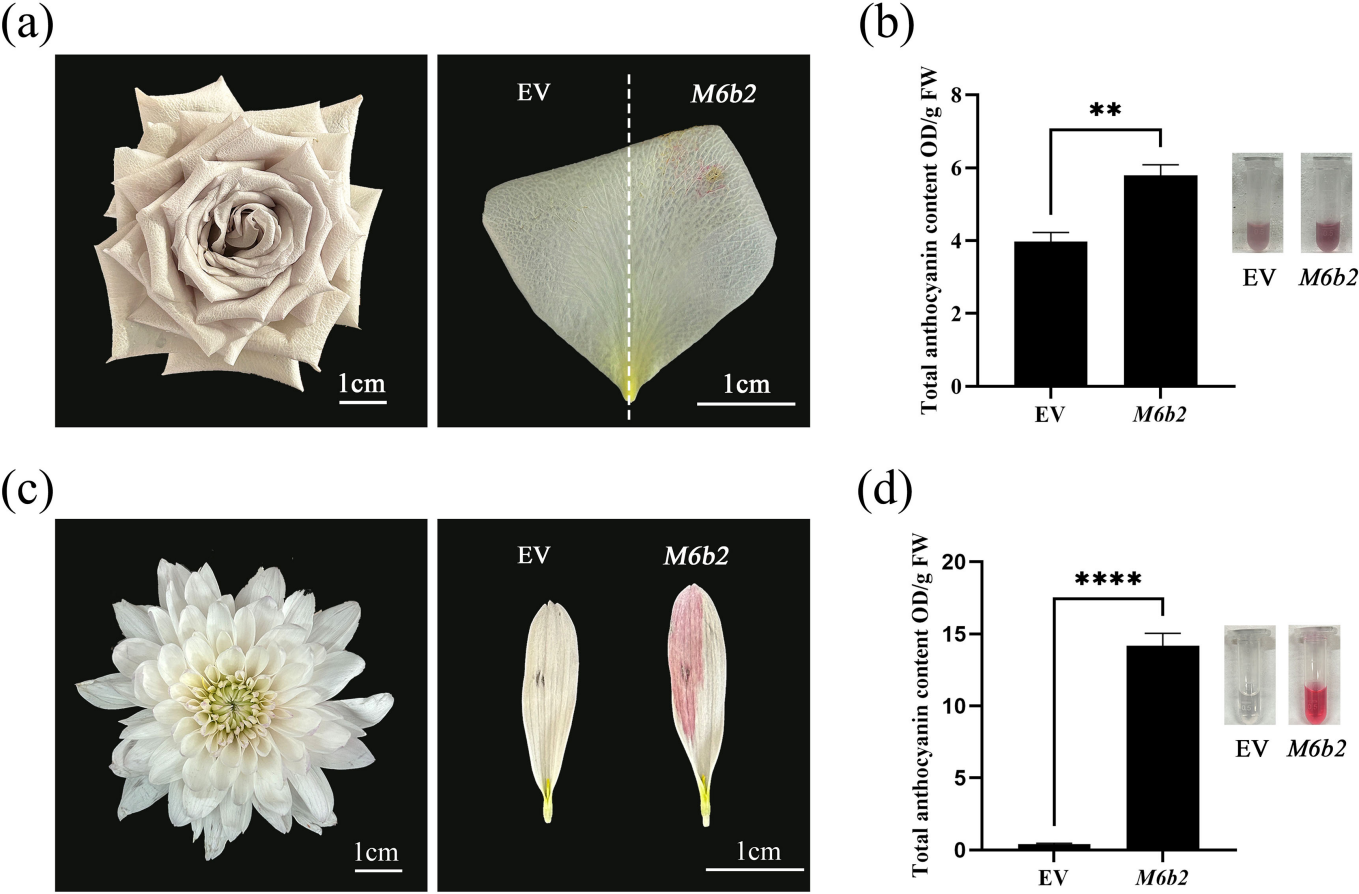
Transient overexpression *35S::CmMYB6-CmbHLH2* induced anthocyanin in rose petals and chrysanthemum florets. **(a)** Phenotypic comparison of rose petals transformed with *35S::CmMYB6-CmbHLH2* and empty vector (EV); **(b)** Comparison of total anthocyanin content in transformed rose petals infiltrated with *35S::CmMYB6-CmbHLH2* and EV; **(c)** Phenotypic comparison of chrysanthemum florets transformed with *35S::CmMYB6-CmbHLH2* and EV; **(d)** Comparison of total anthocyanin content in transformed chrysanthemum florets infiltrated with *35S::CmMYB6-CmbHLH2* and EV; ** indicates a significant difference at P < 0.01; ****Indicates significant correlation at the P ≤ 0.0001 level.

In rose petals, transient expression of the *35S::CmMYB6-CmbHLH2* resulted in a distinct pale-pink pigmentation in the transformed region, whereas the empty vector (EV) control showed no visible color change (**Figure 6a**). Quantification of total anthocyanin content confirmed a significant increase in *35S::CmMYB6-CmbHLH2*-transformed petals, with levels reaching 5.79 OD/g FW, which was approximately 1.5-fold higher than the EV control (**Figure 6b**, P < 0.01).

In chrysanthemum ray florets, transiently over expressed *35S::CmMYB6-CmbHLH2* triggered a striking red pigmentation that was absent in the EV control (**Figure 6c**). The total anthocyanin content in *35S::CmMYB6-CmbHLH2*-transformed florets increased to 14.17 OD/g FW, representing a >100-fold increase relative to the EV control (**Figure 6d**).

Collectively, these results demonstrate that the *CmMYB6-CmbHLH2* complex can efficiently induce anthocyanin accumulation across diverse ornamental plant species, exhibiting functional conservation and potential for broader application in modulating pigmentation in petals, florets and leaves.

## 4 Discussion

### 4.1 Effects of different vectors and *Agrobacterium* strains on visual pigmentation efficiency and the underlying mechanisms

Different vectors and *Agrobacterium* strains had pronounced effects on the efficiency of *CmMYB6-CmbHLH2* mediated visual pigmentation, highlighting the importance of transformation system optimization for reliable phenotypic reporting. Among the tested combinations, the EHA105 strain carrying the SK vector consistently produced the strongest visible pigmentation, highest anthocyanin accumulation, and greatest transgene expression levels, indicating superior delivery and expression efficiency. This enhanced performance may be attributed to multiple factors, including the higher virulence and T-DNA transfer capacity of the EHA105 strain, as well as vector-related characteristics (Hellens et al., 2000). Notably, the pGreenII 0029 62-SK vector has a substantially smaller backbone size (2247 bp) compared with pCAMBIA-1301 (11,849 bp), and shorter plasmids are generally associated with higher transformation and expression efficiency. In contrast, combinations involving the GV3101 strain or the larger 1301 vector resulted in weaker pigmentation and lower expression levels, suggesting limitations in infection efficiency or transgene delivery. The strong positive correlation observed between transgene expression and anthocyanin accumulation further supports the conclusion that both *Agrobacterium* strain selection and vector architecture jointly determine the reliability of anthocyanin-based visual reporters.

### 4.2 Environmental factors differentially regulate *CmMYB6-CmbHLH2* mediated anthocyanin-based visual pigmentation

Environmental conditions had profound but distinct effects on anthocyanin accumulation induced by *CmMYB6-CmbHLH2* complex, indicating that the stability and reliability of this visual reporter system are strongly environment-dependent. Light availability, temperature, and drought stress regulated visible pigmentation through different mechanisms, underscoring the multilayered control of anthocyanin biosynthesis and the necessity of defining appropriate environmental boundaries for reporter system application.

Under dark conditions, *CmMYB6–CmbHLH2*-mediated pigmentation was almost completely abolished, despite the presence of detectable *CmMYB6* and *CmbHLH2* transcripts. Although transcript levels were moderately reduced (42% and 62% downregulation for *CmMYB6* and *CmbHLH2*, respectively), the magnitude of anthocyanin depletion (91% reduction) was far greater than the decrease in gene expression, suggesting that darkness suppresses anthocyanin accumulation predominantly at post-transcriptional or protein levels. A key mechanism underlying this post-transcriptional regulation is likely the ubiquitin-proteasome system (UPS)-mediated degradation of the CmMYB6-CmbHLH2 protein complex, which is tightly controlled by light signaling pathways. For instance, CONSTITUTIVE PHOTOMORPHOGENIC 1 (COP1), a central negative regulator of light signaling in plants (Maier and Ute, 2015; Liu et al., 2023), may mediate the ubiquitination and subsequent degradation of the CmMYB6-CmbHLH2 complex under dark conditions, which would explain why detectable CmMYB6 and CmbHLH2 transcripts fail to drive significant anthocyanin accumulation in darkness-even though the genes are transcribed.

In contrast, high temperature primarily impaired anthocyanin accumulation by strongly suppressing transgene expression. Exposure to elevated temperature (37°C) nearly eliminated *CmMYB6* and *CmbHLH2* transcript (expression 99% and 95% reduction, respectively), resulting in a dramatic loss of visible pigmentation and anthocyanin content (95% reduction). Unlike darkness, where transcriptional repression was partial, high temperature reduced transgene expression to levels comparable with the empty vector control, indicating that temperature stress acts mainly at the transcriptional level. This temperature-dependent transcriptional suppression likely involves multiple interconnected mechanisms: First, heat stress may compromise the activity of the 35S promoter driving CmMYB6-CmbHLH2 expression, as constitutive promoters are often sensitive to temperature fluctuations due to altered chromatin accessibility or transcription factor binding affinity; Second, high temperature can inhibit Agrobacterium-mediated transient transformation efficiency, reducing the number of cells expressing the transgene and thus overall transcript levels; Third, heat stress may accelerate the degradation of transgene transcripts through enhanced activity of RNA-degrading enzymes (e.g., ribonucleases) or impaired mRNA stability. Collectively, these mechanisms converge to abrogate CmMYB6-CmbHLH2 expression, thereby blocking anthocyanin biosynthesis at its transcriptional initiation.

Interestingly, drought stress exerted an opposite effect, moderately enhancing both anthocyanin accumulation and *CmMYB6–CmbHLH2* expression, resulting in a stable and intensified pigmentation phenotype. Given the well-established role of anthocyanins in stress adaptation and reactive oxygen species scavenging, drought-induced upregulation of the anthocyanin pathway likely amplifies the visual output of the reporter system. While this enhancement confirms the robustness of the *CmMYB6–CmbHLH2* reporter under drought conditions, it also suggests that stress-responsive activation of anthocyanin biosynthesis may exaggerate visible signals, which should be carefully considered when interpreting reporter-based phenotypes under abiotic stress conditions.

### 4.3 The advantages and limitations of the *35S::CmMYB6-CmbHLH2* complex as a visual reporter gene in different plants

The present study demonstrates that the *CmMYB6–CmbHLH2* regulatory module can function as an effective anthocyanin-based visual reporter, as visible anthocyanin accumulation mediated by this complex showed a clear positive correlation with transgene expression levels, supporting its feasibility as a qualitative indicator of overexpression efficiency. Compared with conventional reporter systems such as GUS (Bai and DeMason, 2008; Wei et al., 2017; Honma and Yamakawa, 2019; Kim et al., 2010) or fluorescent proteins (Pasin et al., 2014; Kodama and Wada, 2009; Lummer et al., 2013; Chen et al., 2019; Subedi, 2022), the anthocyanin-based reporter offers additional advantages, including direct visual detection without the need for staining procedures or imaging equipment, making it particularly suitable for rapid assessment of transient expression and transformation efficiency in ornamental plants.

However, the reliability of the *CmMYB6–CmbHLH2* visual reporter system is subject to notable limitations. Anthocyanin accumulation is intrinsically influenced by environmental and physiological factors, as demonstrated by the strong dependence on light and temperature conditions observed in this study. In addition, stress-related signals, such as drought, may enhance anthocyanin biosynthesis independently of transgene expression, potentially leading to overestimation of reporter activity. Therefore, while the *CmMYB6–CmbHLH2* based system provides a convenient and intuitive readout, its application requires strict control and standardization of environmental conditions. Defining appropriate usage boundaries and integrating complementary molecular analyses are essential to ensure accurate interpretation of visible pigmentation when employing this reporter system.

The conservation of the CmMYB6-CmbHLH2 complex in different plant species is a key factor determining its applicability as a universal visual reporter. At present, the plant species we have tested for the CmMYB6-CmbHLH2 complex-mediated anthocyanin accumulation include Arabidopsis thaliana, Nicotiana tabacum, Rosa chinensis, Chrysanthemum morifolium, and Lilium brownii. Our results show that the CmMYB6-CmbHLH2 complex can conservatively regulate anthocyanin synthesis in four of these species (Arabidopsis thaliana, Nicotiana tabacum, Rosa chinensis, and Chrysanthemum morifolium), consistently inducing visible anthocyanin accumulation and activating the expression of anthocyanin biosynthetic genes. However, this regulatory effect was not observed in Lilium brownii. The failure of the CmMYB6-CmbHLH2 complex to induce anthocyanin accumulation in Lilium brownii may be attributed to several potential mechanisms: first, the function of the CmMYB6 and CmbHLH2 genes may not be conserved in Lilium brownii, leading to the loss of their ability to activate the anthocyanin biosynthetic pathway; second, Lilium brownii may lack key structural genes or regulatory factors required for anthocyanin synthesis, resulting in the inability to respond to the CmMYB6-CmbHLH2 complex; third, the endogenous regulatory network of Lilium brownii may have unique characteristics that interfere with the function of the CmMYB6-CmbHLH2 complex, thereby blocking anthocyanin accumulation. Further studies are needed to clarify the specific molecular mechanisms underlying this species-specific difference.

## Data Availability

The raw data supporting the conclusions of this article will be made available by the authors, without undue reservation.

## Supplementary material

Supplementary Figure 1 Phenotypic comparison of empty vector (EV) controls and *35S::CmMYB6-CmbHLH2* overexpression in different vector-strain combinations On the left side of the leaf is the empty vector control, and on the right side is the *35S::CmMYB6-CmbHLH2.*
