# Supplementary figures and images for: Environmental dependence of visible anthocyanin accumulation mediated by the CmMYB6–CmbHLH2 complex
Source: Front Plant Sci. 2026 May 1;17:1800973. doi: 10.3389/fpls.2026.1800973 (PMC13176266; doi:10.3389/fpls.2026.1800973)

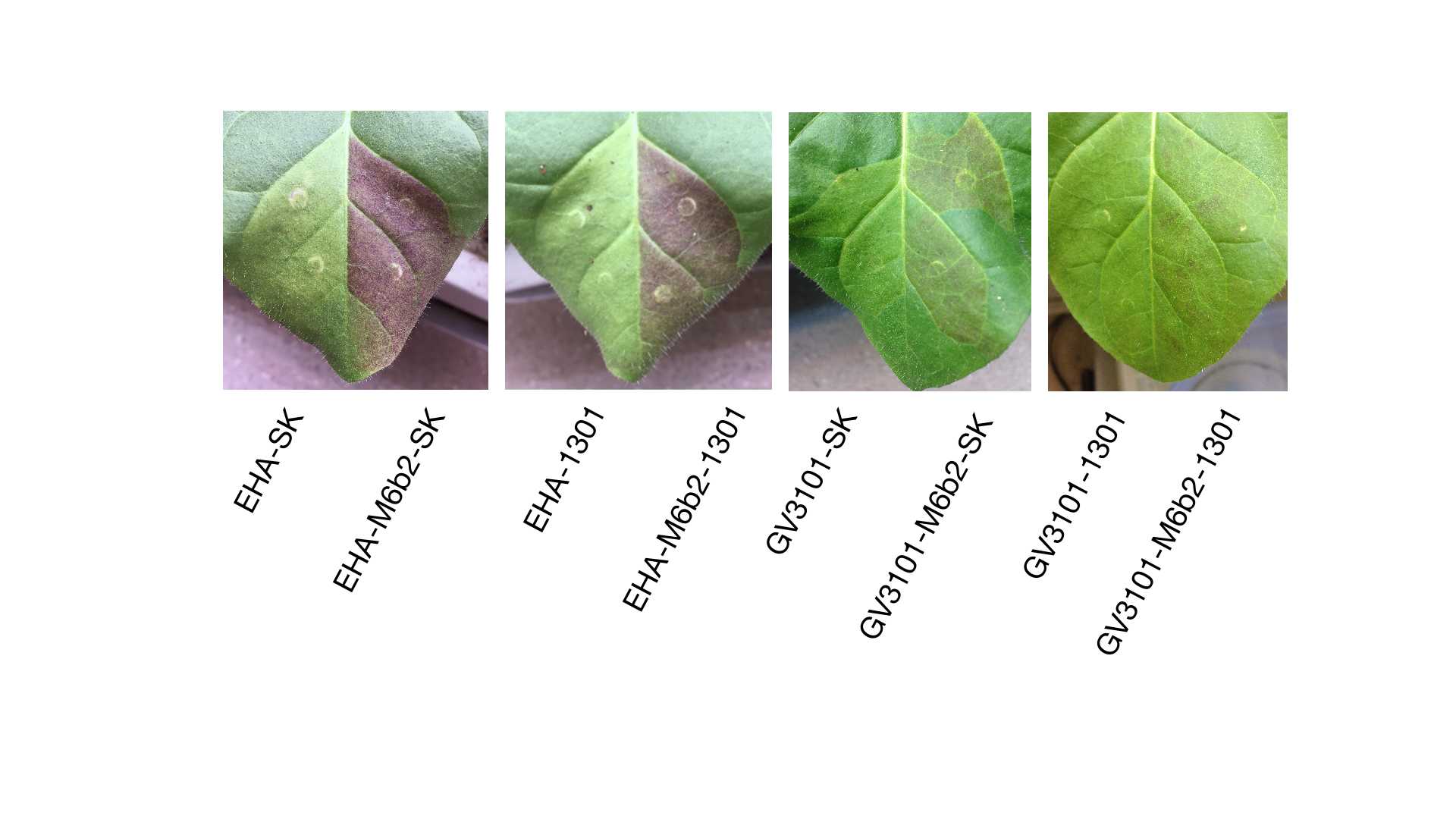

Supplement: Supplementary Figure 1 — Phenotypic comparison of empty vector (EV) controls and 35S::CmMYB6-CmbHLH2 overexpression in different vector-strain combinations On the left side of the leaf is the empty vector control, and on the right side is the 35S::CmMYB6-CmbHLH2. [file Image1.jpeg]
